# Microbial-derived metabolites as a risk factor of age-related cognitive decline and dementia

**DOI:** 10.1186/s13024-022-00548-6

**Published:** 2022-06-17

**Authors:** Emily Connell, Gwenaelle Le Gall, Matthew G. Pontifex, Saber Sami, John F. Cryan, Gerard Clarke, Michael Müller, David Vauzour

**Affiliations:** 1 Faculty of Medicine and Health Sciences, Norwich Medical School, University of East Anglia, Norwich, NR4 7TJ UK; 2 APC Microbiome Ireland, University College Cork, Cork, Ireland; 3 Department of Anatomy and Neuroscience, University College Cork, Cork, Ireland; 4 Department of Psychiatry and Neurobehavioural Science, University College Cork, Cork, Ireland

**Keywords:** Microbiota-gut-brain axis, Alzheimer’s disease, Brain, TMAO, Tryptophan, Bile acids, Cresols, Indoles

## Abstract

A consequence of our progressively ageing global population is the increasing prevalence of worldwide age-related cognitive decline and dementia. In the absence of effective therapeutic interventions, identifying risk factors associated with cognitive decline becomes increasingly vital. Novel perspectives suggest that a dynamic bidirectional communication system between the gut, its microbiome, and the central nervous system, commonly referred to as the microbiota-gut-brain axis, may be a contributing factor for cognitive health and disease. However, the exact mechanisms remain undefined. Microbial-derived metabolites produced in the gut can cross the intestinal epithelial barrier, enter systemic circulation and trigger physiological responses both directly and indirectly affecting the central nervous system and its functions. Dysregulation of this system (i.e., dysbiosis) can modulate cytotoxic metabolite production, promote neuroinflammation and negatively impact cognition. In this review, we explore critical connections between microbial-derived metabolites (secondary bile acids, trimethylamine-N-oxide (TMAO), tryptophan derivatives and others) and their influence upon cognitive function and neurodegenerative disorders, with a particular interest in their less-explored role as risk factors of cognitive decline.

## Background

Age is the predominant risk factor for cognitive decline. Whilst some decline in cognition is considered an inevitable part of healthy ageing, deleterious changes in cognition, including mild cognitive impairment (MCI) and age-related dementias (e.g., Alzheimer’s disease, AD), are estimated to impact approximately 15% and 11% of the population over 65 years respectively [1, 2]. By 2050, the global elderly population is expected to increase by 21% [2], increasing incidences of cognitive decline [3]. Cognitive decline exacerbates broad social and economic issues, including depression, social withdrawal, difficulties performing everyday tasks, drastic reductions in quality of life and greater reliance on others (social care) [4]. Understanding how to promote healthy ageing whilst resisting aberrant changes in cognition is therefore becoming a priority.

**Fig. 1:**
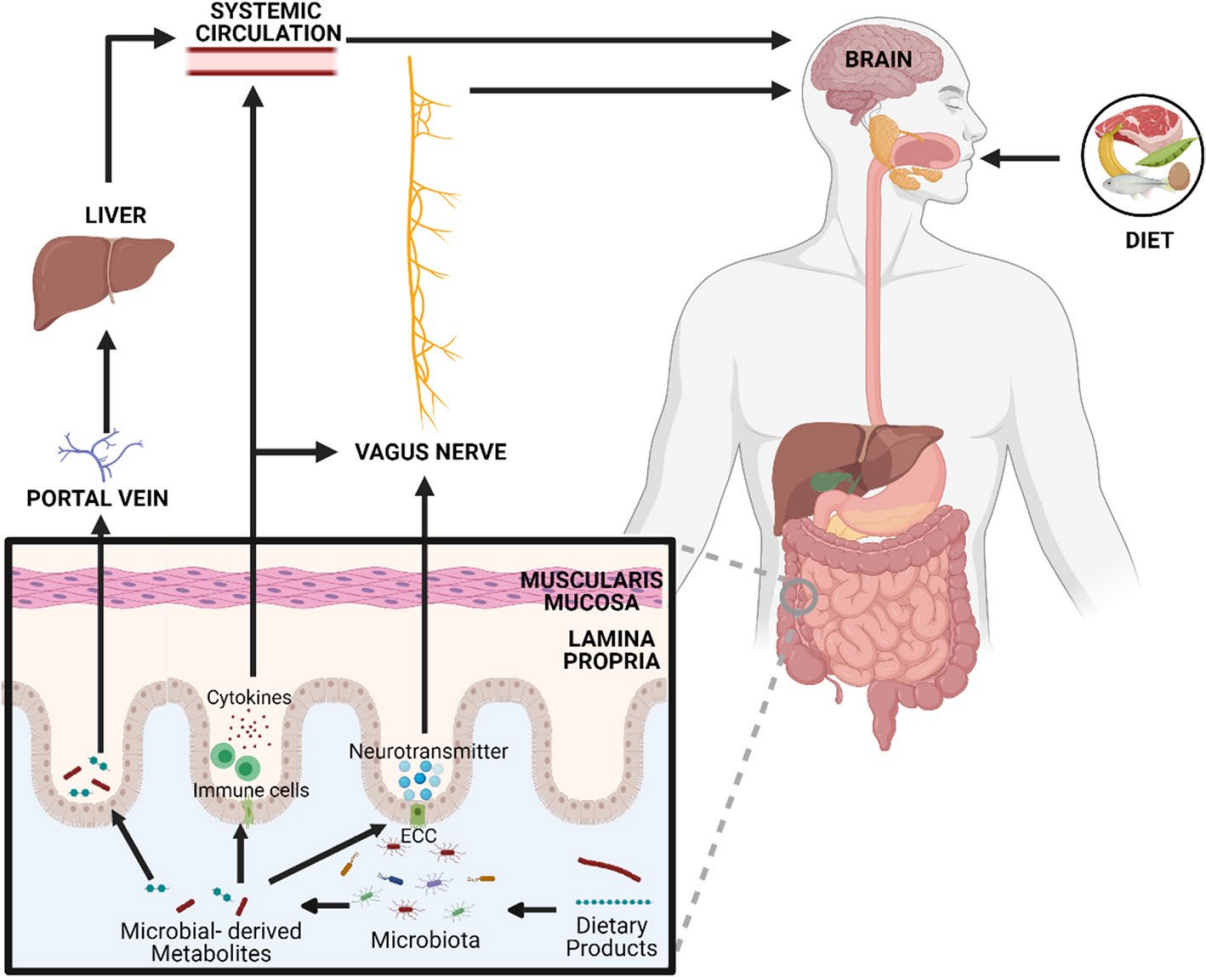
Microbial metabolites can directly and indirectly modulate the CNS through immune, neuronal and direct metabolite mediated pathways within the microbiota-gut-brain axis. In the gut lumen, dietary products can be metabolised by microbiota into neuroactive compounds, including neurotransmitters, (e.g., serotonin, dopamine), amino acids (e.g., tryptophan, tryptamine) and other microbial-derived metabolites (e.g., short-chain fatty acids, trimethylamine (TMA)). These compounds subsequently communicate with the central nervous system either directly, travelling through the portal vein, liver and crossing the blood–brain barrier, or indirectly via the production of neurotransmitters by enterochromaffin cells (ECC) or immune pathways (stimulated immune cells produce cytokines that can enter the blood or stimulate the vagus nerve)

Addressing modifiable risk factors can delay the onset, or even ameliorate cognitive decline [5], whilst assisting with the identification of asymptomatic individuals with an increased chance of developing the condition in the future [6]. Currently, hypertension [7], diabetes mellitus [8, 9], arteriosclerosis [10], obesity [11] and hypercholesterolemia [12] are the most significant risk factors associated with age-related cognitive decline among others [13]. Given the connection between cognition and these metabolic diseases, it is perhaps unsurprising that dietary factors can elicit a substantial influence upon cognitive function [14] through the modulation of a microbiota-gut-brain axis [15]. The microbiota-gut-brain axis is a complex communication system bridging the gut, liver and the central nervous system (CNS) that is modulated by the microbiome, a collection of 10^14^ microorganisms with an extensive functional gene repertoire [16]. These microorganisms predominantly reside in the gut, metabolising dietary compounds into a vast range of metabolites. Metabolites can cross the intestinal epithelial barrier; a structure connected by tight junction proteins, lamina propria and reinforced by mucosal secretions [17], primarily via active transport, and enter systemic circulation. From here, metabolites can directly initiate physiological responses by crossing the blood–brain barrier (BBB) and influencing the CNS [18], or indirectly via vagus nerve stimulation (Fig. 1) [19].

The capability of microbial-derived bioactive metabolites to influence the CNS provides a novel mechanistic pathway for cognitive decline, warranting its further exploration. Within the gastrointestinal (GI) tract, microbiota populations are in part reflective of their local physiological conditions. The small intestine, due to its proximity with the stomach, contains high concentrations of acids, oxygen and antimicrobials, thereby restricting bacterial growth to predominantly fast-growing anaerobes that can adhere to epithelia or mucus [20]. Conversely, colonic regions promote much denser bacterial communities, dominated by anaerobes such as *Prevotellaceae* and *Lachnospiraceae*, that can digest complex carbohydrates [21]. Numerous intrinsic factors (e.g., genetics, immune response, metabolites) and extrinsic factors (e.g., diet, lifestyle) also impact gut microbial composition, making it an attractive therapeutic intervention target [22]. The composition of these microbial communities determines the concentration of neurotransmitters or neuromodulators (including microbial-derived metabolites) released into circulation. Broad deviations in these microbial compositions, often referred to as “dysbiosis”, condition distinctly different metabolic profiles that may contribute to cognitive decline [23, 24]. Gut microbial composition is known to be significantly altered in patients with MCI, a transitional stage preceding AD, suggesting microbial changes may occur in the early stages of cognitive decline and influence its progression [25–29].

Intestinal microbiota possess the capacity to produce hundreds of metabolites [30, 31], yet the influence of these compounds on cognitive health have not been uncovered. The present review details the roles of newly emerging microbial-derived metabolites that are less-explored in the current literature in the context of cognitive health and provide an additional in-depth discussion of their use as potential indicative factors of cognitive decline.

## Age-related cognitive declines

As we age, some of our cognitive abilities decline. Cognitive capabilities such as verbal skills, remain largely unaffected by brain ageing and can even improve over time [32]. Other essential capabilities, including mental reasoning, memory (in particular episodic, working and recognition memory) and processing speed, steadily deteriorate with age (See review [33] for further information). During ageing, the brain undergoes various structural and functional changes. The most apparent being a gradual shrinkage of the brain, alongside an increase in ventricular space and cerebrospinal fluid (CSF) [34, 35]. Brain atrophy increases in the elderly in an anterior–posterior gradient, with the most severe consequences taking place in the prefrontal regions [36, 37]. A reduction in white matter (the nerve fibres connecting different brain regions) integrity has been linked with normal cognitive ageing, impairing the transfer of information between cortical regions [38], an essential process for higher cognitive functioning [39].

Structural neuroimaging highlights differing trends in the neurobiology of pathological ageing and detrimental cognitive decline. Here, individuals are more likely to experience reductions in gray matter in the dorsolateral and medial prefrontal, parietal, and lateral temporal regions [40, 41], alongside a loss of white matter integrity in the cingulum, corpus callosum, and superior longitudinal fasciculus [42–44]. This is instead of a decline in the frontal regions that typically occurs in healthy ageing.

AD is also associated with volume loss in the medial temporal lobe, a brain region highly associated with memory functions. Reduction typically starts in the anterolateral entorhinal cortex and advances medially across the remaining entorhinal cortex to the hippocampus [45, 46], with atrophy occurring at rates of 4.9–8.2%. In healthy ageing, atrophy in these regions occurs at a lower rate, diverging from pathological ageing, at 0.2–3.8% [46]. More recently, using longitudinal MRI and PET data, a similar divergence in volume loss has been noted in the locus coeruleus [47].

Finally, the default mode network (DMN), a resting-state network associated with cognitive processes of oneself (e.g., autobiographical memory), demonstrates connectivity patterns that distinguish healthy ageing from AD. Results from a task free-fMRI suggest AD patients have an accelerated ageing pattern of connectivity [48] and decreased resting-state activity in the posterior cingulate and hippocampus when compared with age-matched controls [49]. However, the biological mechanisms behind the heterogeneity of age-related cognitive decline are complex and not well understood.

## The microbiota-gut-brain axis in the context of ageing and cognitive decline

The human gut microbiome represents a complex community of microbes that live in a mutualistic relationship with their host. Initially, these microorganisms were considered to be solely responsible for intestinal processes (fermentation of carbohydrates, synthesis of vitamins and xenobiotic metabolism) [50]. However, over the last 15 years, this notion has been revised, owing to increasing evidence of a bidirectional communication system between the CNS and the GI tract, more commonly referred to as the ‘gut-brain axis’.

The gut-brain axis encompasses the CNS, the autonomic and enteric nervous system, and peripheral nerves and is vital for maintaining homeostasis. Signals from the brain control the secretory and sensory function of the gut, whilst the brain and gut communicate via physiological channels including the neuroendocrine, autonomic nervous system, neuroimmune pathways and molecules synthesised from gut microbes [51]. Since the gut microbiota is integral to the modulation of this communication at different levels (from the gut lumen to the CNS) and chronologically as we age, many have broadened the term to ‘microbiota-gut-brain axis’ [52]. Indeed, the existence of the microbiota-gut-brain axis is supported by substantial preclinical and human evidence, highlighting its effect on different cognitive domains. Firstly, germ-free (GF) mice show that the brain is markedly affected by the absence of microbiota, exhibiting deficiencies in learning, memory recognition and emotional behaviours [53–56]. These behavioural changes were accompanied by altered brain-derived neurotrophic factor (BDNF) expression in the hippocampus [54, 57, 58], a molecule inherently linked with synaptic plasticity and cognitive function [59–61], and significant microbiota-associated changes in the quantity of dopamine and activation of serotonin synthesis pathways [62–65], suggesting an important role of microbiota in memory, brain health and behaviour. Secondly, chronic antibiotic depletion of microbiota populations alters tryptophan metabolism and the expression of key cognitive signalling molecules in the brain such as BDNF, N-methyl-d-aspartate receptor subunit 2B (NR2B), serotonin transporter, neuropeptide Y system, oxytocin and vasopressin [66, 67]. These changes are associated with long-lasting effects on cognition and increases in anxiety-related behaviours [66, 68]. Finally, administering specific prebiotics/probiotics modulates behaviour in both rodents and humans, including changes in depression, anxiety and stress [57, 69–73], alongside changes in immune markers, hippocampal synaptic efficacy and tryptophan metabolism [74, 75].

As we age, microbiota composition and function changes [76]. In humans, this has been associated with a decrease in species diversity, a reduction in Clostridiales and *Bifidobacterium* and a rise in Proteobacteria and pathobionts such as Enterobacteriaceae [76, 77]. However, abnormal alterations in intestinal microbiota composition, as seen in early cognitive decline and AD [25, 78], are associated with local and systemic inflammation, and dysregulation of the microbiota-gut-brain axis [79]. Advances in sequencing technologies have enabled us to investigate the association between cognitive decline and gut dysbiosis at the phylum level. These studies have highlighted differences in taxonomic levels of Bacteroides, Firmicutes, Actinobacteria, Ruminococcus, Lachnospiraceae, and Selenomonadales between AD patients in comparison to controls [25, 78, 80–82].Such dysregulation has been associated with an increase in inflammatory markers, cytokines and the permeability of the gut epithelial barrier (‘leaky gut’), resulting in excessive leakage of bioactivate molecules, such as short-chain fatty acids (SCFAs), kynurenines, melatonin, histamine, bile acids, and neurotransmitters, into the blood. The resulting increase in neuroactive products can no longer efficiently be removed by the body’s next barrier; the liver, and therefore can cause a variety of physiological changes directly and indirectly affecting the CNS, including further decrease in BBB function. In the elderly population, this dysregulation becomes particularly relevant, as the BBB becomes more permeable with age [83]. A more permeable BBB allows an increased influx of harmful blood components, including microbial metabolites, into the brain; a feature seen in AD patients (reviewed by [84]). This process promotes neuroinflammation and macrophage dysfunction, leading to neural injury and ultimately a reduction in cognitive function [85, 86].

As previously outlined, the gut can also influence the brain indirectly through the vagus nerve activation. The vagus nerve consists of 80% afferent and 20% efferent fibres [87]. Afferent fibres connect to all four layers of the digestive tract, but do not cross the epithelial layer and therefore are not in direct contact with gut microbiota. As a result, the microbiota activates these fibres indirectly via the release of metabolites or bacterial products. Enteroendocrine cells (ECCs) make up approximately 1% of intestinal epithelial cells and can detect signals from the microbiota through toll-like receptors (TLR), capable of identifying bacterial compound such as lipopolysaccharides (LPS) [88], or through receptors activated by microbiota-derived metabolites such as SCFAs [89]. ECCs can subsequently interact with vagal afferent fibres through the release of serotonin or gut hormones [90, 91]. This indirect signalling between the gut microbiota and the brain via the vagus nerve can modulate certain cognitive functions. For example, rodents fed with the probiotic *L. rhamnosus* for 28 days had a decrease in anxiety-related behaviour, whilst inducing region-dependent alterations in γ-aminobutyric acid (GABA) receptor [92]. Importantly, this result only occurred with an intact vagus nerve, as mice undergoing a vagotomy did not display these behavioural and neurochemical changes. Similarly, in a colitis model, the normalisation of anxiety-like behaviours by the probiotic *Bifidobacterium longum* NCC3001 was found to be vagally dependent [93]. However, the total effects of the microbiome are not solely dependent on the vagus nerve stimulation, as mice orally receiving a mixed antimicrobial treatment had altered exploratory behaviour and hippocampal BDNF, independently of vagal integrity [94]. Together, these data emphasise that while the vagus nerve provides a crucial bridge allowing communication between the gut, its microbiome and brain, there are also other essential routes of communication comprising the microbiota-gut-brain axis, indicating its complex connectivity.

## The microbiota-gut-liver-brain axis

The relationship between the gut, liver and brain has increasingly been highlighted in recent years due to a high prevalence of liver disease, which is commonly accompanied by clear and global cognitive impairment (hepatic encephalopathy) [95, 96]. The gut and liver are linked by the portal vein, biliary tract and systemic circulation, allowing microbial and host-derived metabolites to influence liver function. Conversely, the liver acts as a vital barrier, removing potentially harmful compounds from the blood using a range of hepatic immune cells, including Kupffer cells, hepatic stellate cells and natural killer cells [97], modulating the concentration of metabolites directly and indirectly influencing the CNS. The liver also controls unrestricted bacterial growth in the gut, maintaining gut eubiosis, through the transport of bile salts through the biliary tract into the intestinal lumen leading to the secretion of antimicrobial compounds [98, 99]. For example, bile acids can bind to the FXR receptor in enterocytes, initiating the production of antimicrobial peptides such as angiogenin 1 and RNAse family member 4, which can inhibit bacterial overgrowth in the gut and intestinal barrier dysfunction [100]. Gut dysbiosis causes an imbalance of microbial and host-derived products, reducing the epithelial barrier function and causing increased leakage in the system. Long-term, this process can initiate metabolic disorders in the liver, promoting liver damage (reviewed by [101]). As such, liver damage has been found to correlate with the severity of gut dysbiosis [102]. Since a diseased liver cannot effectively remove harmful products from the blood or inhibit the overgrowth of bacteria [103–105], this process can accelerate microbiota-gut-brain axis dysregulation and ultimately cognitive decline. Thus, when considering the occurrence of cognitive decline associated with microbial-derived metabolites, the role and function of the liver cannot be ignored.

## Microbial-derived metabolites and cognitive decline

### Bile acids

**Fig. 2:**
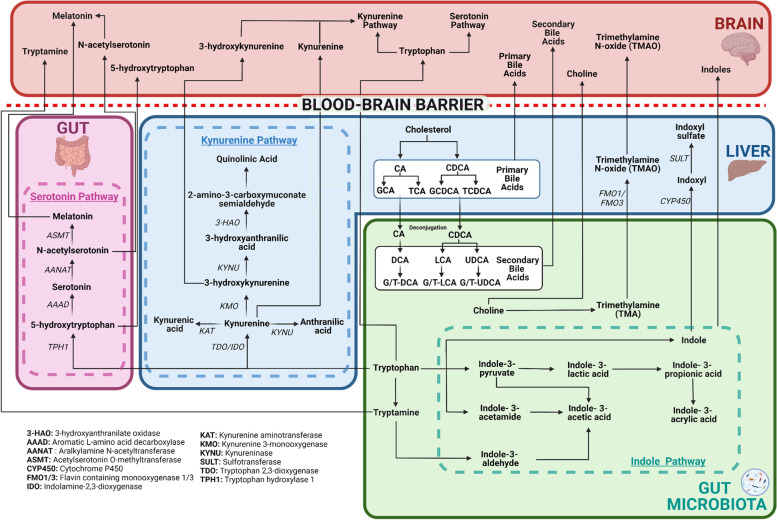
Bile acids, TMAO and tryptophan metabolic pathways and their links to the brain. Primary bile acids are produced from cholesterol breakdown in the liver. They can be conjugated with taurine or glycine residues before travelling to the gut, where they are deconjugated and converted to secondary bile acids via microbial action. Bile acids have been found in the brain of humans and rodents suggesting they can cross the blood–brain barrier via either diffusion (unconjugated) or active transport (conjugated) and influence the central nervous system. TMAO is produced via a two-stage process. TMA is first formed from the microbial conversion of choline in the gut. TMA then travels to the liver, where the FMO1/3 enzyme converts it to TMAO. Recent evidence found TMAO in human brains, indicating it can cross the blood–brain barrier. Tryptophan can be metabolised via three key pathways. Firstly, via gut microbial action, tryptophan can be converted via the indole pathway into numerous indole derivatives, or into the amino acid, tryptamine. Indoles and tryptamine are known to cross the BBB. Secondly, around 3% of dietary tryptophan is metabolised into serotonin and melatonin via numerous enzymes in the serotonin pathway. Notably, serotonin produced in the gut cannot cross the blood–brain barrier. However, the serotonin precursor, 5-hydroxytryptophan, and serotonin derivatives, N-acetylserotonin and melatonin, can cross the blood–brain barrier and influence the central nervous system. Finally, the majority of tryptophan (~ 90–95%) is metabolised via the kynurenine pathway, of which 90% occurs in the liver. This pathway is initiated by the TDO enzyme in the liver and the IDO enzyme in the brain. Only kynurenine, 3-hydroxykynurenine and tryptophan itself can cross the blood–brain barrier. However, once in the brain, tryptophan can be metabolised via both the kynurenine and serotonin pathways to form the pathway’s intermediates

Humans produce large, hydrophilic pools of primary bile acids (BA) from cholesterol in the liver that are secreted into bile (Fig. 2). BAs are largely synthesised via two biosynthetic pathways: the classical pathway and the alternative pathway [106]. The classic pathway produces the majority of BAs in humans (~ 90%) and is initiated by the cholesterol 7α-hydroxylase (CYP7A1) enzyme to synthesise the primary BAs cholic acid (CA) and chenodeoxycholic acid (CDCA) [107]. The alternative pathway contributes less than 10% of BA synthesis (with more minor pathways contributing the remainder) and is initiated by sterol 27-hydroxylase (CYP27A1) [108]. After synthesis in the liver, CA and CDCA can be conjugated with hydrophilic taurine or glycine residues before they are secreted from hepatocytes into the bile canaliculi. They are stored in the gallbladder ready to be distributed into the small intestine following a meal to expedite digestion and emulsify dietary lipids and fat-soluble vitamins. Once secreted into the small intestine, more hydrophobic secondary BAs are formed by gut bacteria and are subsequently excreted or reabsorbed in the ileum to enter the enterohepatic circulation and recycle back to the liver [109]. This efficient process ensures BAs are recycled between 4 to 12 times a day [106].

In the brain, cholesterol can be metabolised by a final pathway known as the neural cholesterol pathway. As the brain is one of the more sensitive organs to hypercholesterolemia, this cholesterol breakdown is essential to maintaining brain health [110]. Excess cholesterol becomes oxidised into 24- and 25-hydroxycholesterol by cholesterol 24-hydroxylase (CYP46A1), an enzyme primarily expressed in the brain [111]. Once 24(S)-hydroxycholesterol is formed, it can pass through the BBB and enter circulation. From here, 24(S)-hydroxycholesterol travels back to the liver to be metabolised by CYP39A1 and continue in BA synthesis [111]. In mice with mutated CYP46A1 function, 24(S)-hydroxycholesterol is not formed and is associated with impairments in spatial, associative and motor learning, highlighting the importance of this pathway for maintaining cognitive function [112].

### BAs in the brain

Over 20 conjugated and unconjugated BAs and their receptors have been reported in both human and rodent brains [110, 113–115], suggesting BAs can not only cross the BBB but also bind to nuclear receptors and initiate physiological responses [115, 116]. However, the mechanism by which BAs cross the BBB is still uncertain. Unconjugated BAs may diffuse across the BBB as CA, CDCA and deoxycholic acid (DCA) are known to diffuse across phospholipid bilayers [117] and concentrations in the brain correlate with serum levels [113]. On the other hand, conjugated bile salts must cross the BBB via active transport due to their hydrophilic anionic structure at physiological pH [118, 119]. Indeed, members of the solute carrier (SLC) family, such as the organic anion transporting polypeptides (OATP1A4 and 1C1) [120] and the apical sodium-dependent bile acid transporter (ASBT or SLC10A2) [121], and members of the ATP-binding cassette transporters (ABC) family such as ABCC2 and ABCC4 [122, 123] have been identified in the brain. Conversely, Baloni and colleagues through a large-scale transcriptomics analysis of 2,114 post-mortem brains identified only three BA transporters (ABCC1, ABCC4 and SLC51A/SLC51B) in the brain [110]. The primary role of these transporters is to reduce the concentration of cytotoxic molecules by transporting them into the bloodstream [124]. Yet, since these transporters occur on both the basolateral (blood-facing) and apical (brain-facing) side [125], they may also transport molecules into the CNS from systemic circulation, indicating a potential endogenous signalling role of BAs in the brain. However, there is still a lack of direct evidence of in vivo transport of BA over the BBB [125].

### BAs and cognitive function

While BA function in the GI tract is well-characterised, significantly fewer studies investigate their effect in the brain, limiting our knowledge [111]. Accumulating evidence suggests that cognition can be influenced by the dysregulation of BA synthesis and metabolism. Indeed, BAs profiles are reportedly altered in cases of MCI and AD, with an increase in cytotoxic secondary BAs and a decrease in primary BAs, suggesting a role of the gut microbiome in the disease progression [126]. Specifically, increased serum concentrations of the secondary BA DCA have been observed in AD patients. DCA has been previously linked with the presence of cognitive symptoms [127] and can modulate mitochondrial pathways causing apoptosis in a variety of tissues and cell types [128]. BA dysbiosis, resulting from either liver or microbiota dysfunction, has been subsequently linked to changes in gut permeability, possibly through FXR and TGR5 receptor signalling, and inflammation, promoting further bacterial dysbiosis in the gut [129]. Inflammation is also a known trigger of microglial activation and reduced neuroplasticity [130], possibly through the production of reactive oxygen species [131], highly reactive chemical molecules that have been previously linked with cognitive decline and AD [132, 133]. Although, some have proposed an important physiological role of ROS in brain metabolic signalling [134].

Alternatively, some BAs have been reported to have neuroprotective effects in the brain (for summary see Table 1). The primary BA CA has been identified as an LXR ligand, which in turn promoted midbrain neural development and neurogenesis in zebrafish [135]. Tauroursodeoxycholic acid (TUDCA), a secondary conjugated BA, can suppress amyloid- β (Aβ) -induced apoptosis in neuronal cell cultures and rodent neurons through the inhibition of the E2F-1/p53/BAX pathway [136, 137]. Similarly, in APP/PS1 double-transgenic mice, providing a TUDCA enriched diet for 6 months reduced Aβ aggregates, neuronal apoptosis, memory deficits and phosphorylation of TAU [138–140]. TUDCA has also been shown to induce anti-inflammatory effects in a mouse model of acute neuroinflammation through its binding and activation of G protein‐coupled bile acid receptor 1/Takeda G protein‐coupled receptor 5 (GPBAR1/TGR5), a receptor expressed on microglia [141]. Finally, in adult rats, TUDCA also enhanced neural stem cell proliferation and early neurogenesis [142], processes that are significantly diminished in AD (reviewed by [143]), with some research suggesting increasing neurogenesis may counteract AD pathological outcomes. Together these findings provide convincing evidence that cognition can be influenced by BAs. Yet further research is required to determine the involvement of specific BA transporters and receptors, as well as the subsequent mechanisms in their neuroprotective and detrimental effects.

**Table 1:** Bile acids and their impact on cognition and dementia

Bile Acid	In Vitro/ In Vivo (species)	Model	Findings	Reference
**CA (Primary Bile Acid)**	In Vivo (Male Sprague-Drawly rats)	Ibotenic Acid-Induced Dementia Model	A combination of administering baicalin, jasminoidin and cholic acid improved cognitive performance through the promotion of pathways related to neuroprotection and neurogenesis	[144]
	In Vivo* (Zebrafish)*	Zebrafish embryos exposed to a cholic acid-treated medium	Cholic acid was identified as a new Lxr ligand, which in turn promoted neural development and neurogenesis in the midbrain of zebrafish	[137]
**CDCA (Primary Bile Acid)**	In Vivo* (*Adult male Wistar rats*)*	AlCl_3_ induced AD	CDCA treatment reduces neurotoxicity and cognitive decline via increased insulin signalling	[145]
	In Vitro	Primary dissociated cultures of the posterior hypothalamus	CDCA is an antagonist for NMDA and GABA_A_ receptors and can significantly reduce neuronal firing	[146]
**TCA (Primary Conjugated Bile Acid)**	In Vivo (human)	Human brain tissue with AD pathology vs age-matched healthy controls	TCA was significantly lower (*p* = 0.01) in AD patients than in age-matched controls	[117]
**DCA (Secondary Bile Acid)**	In Vitro	BCS-TC2 human colon adenocarcinoma cells	DCA modulates mitochondrial pathways causing apoptosis	[130]
	In Vivo (human)	Serum samples from AD patients, amnesic MCI patients and healthy controls	DCA was increased in amnesic MCI and AD in comparison to healthy controls and correlated with cognitive symptoms	[129]
**LCA (Secondary Bile Acid)**	In Vivo (human)	Plasma samples from patients with AD, MCI and healthy controls	LCA was significantly higher in AD patients (*p* = 0.004) compared to healthy controls	[147]
**UDCA (Secondary Bile Acid)**	In Vitro	BV-2 microglial cell line	UDCA can initiate an anti-inflammatory effect by inhibiting NF-κB activation	[148]
**TUDCA (Secondary Conjugated Bile Acid)**	In Vitro	Neuron cell cultures and primary rat neurons	Inhibition of the E2F-1/p53/Bax pathway, leading to suppression of Aβ-induced apoptosis	[138]
	In Vitro	Primary cultures of rat cortical and hippocampal neurons	Reduction in synaptic deficits induced by Aβ through inhibiting the downregulation of postsynaptic density protein-95, leading to a reduction in neuronal death	[149]
	In Vivo (*mouse*)	AD model: APP/PS1 double transgenic mice	Dietary TUDCA provided for 6 months decreased Aβ aggregation and enhanced memory retention	[141]
	In Vivo* (mouse)*	AD model: APP/PS1 double transgenic mice	Dietary TUDCA provided for 6 months decreased hippocampal and prefrontal amyloid deposition and inhibited spatial, recognition and contextual memory deficiencies	[150]
	In Vivo* (mouse)*	AD model: APP/PS1 double transgenic mice	Intraperitoneal injections of TUDCA decreased Aβ deposition, glycogen synthase kinase 3β activity, phosphorylation of τ, and neuroinflammation	[142]
	In Vitro	Aβ-treated primary rat cortical neurons	TUDCA prevented Aβ induced cytochrome c release and neuronal death through the PI3K signalling pathway	[151]
	In Vitro	Aβ-treated primary rat cortical neurons	TUDCA reduced Aβ induced apoptosis through the binding to mineralocorticoid receptors	[140]

### BA as a risk factor of cognitive decline

The association between BAs and cognitive decline, in particular with known AD pathologies [152], has raised speculations that BA profiles could be used as a risk factor of cognitive decline. Currently, there is limited research into the topic. However, Olazarάn and colleagues investigated a large cohort of patients with MCI and AD and identified DCA as being independently associated with the presence of cognitive symptoms [127]. Mapstone et al. identified seven blood-based markers which included glycoursodeoxycholic acid (GUDCA) and could predict the onset of AD or amnestic MCI with 2–3 years with an accuracy of over 90% [153]. Similarly, Marksteiner and colleagues were able to differentiate between healthy controls and AD patients from the concentration of lithocholic acid (LCA) in plasma [154]. However, it should be noted this study utilised a relatively small sample size (*n* = 80) and did not control for the effects of varied diets between individuals, warranting further investigation into the use of BAs as risk factors of cognitive decline.

## TMAO

Trimethylamine N-oxide (TMAO) is a microbial-dependent metabolite generated by the breakdown of dietary fish, meat and fat [147, 155]. Trimethylamine (TMA), the precursor to TMAO, is produced from the metabolism of choline, L-carnitine and phosphatidylcholine by anaerobic microbes in the gut, predominantly located in the small intestine (Fig. 2) [144, 145]. TMA subsequently travels through the portal vein to the liver where it is oxidised by flavin-containing monooxygenase 1 and 3 (FMO1 and FMO3) to form TMAO [146]. Once formed, TMAO can enter the systemic circulation, hence TMAO plasma levels (typically 3 μmol/L in healthy individuals [148]) have been found to correlate with the gut microbial composition [149].

### TMAO and the brain

In vivo studies have identified TMAO in the CSF of both mice and humans, implying that circulating TMAO can influence the CNS [147, 150]. The high concentrations of TMAO detected in the human CSF suggests liver-derived TMAO can cross the BBB, however, the penetration mechanism is unclear [151]. It is also possible a portion of TMAO found in the brain may be synthesised de novo, as FMO3, the enzyme required to convert TMA to TMAO, has been detected in the adult brain [156].

### TMAO and cognitive decline

Over the last decade, TMAO has received increased attention in medical studies due to its links with cardiovascular diseases [157], obesity, diabetes [158], chronic kidney disease [159], metabolic syndrome [160], brain ageing and cognitive impairment [161] and neurodegenerative disorders such as AD [147]. However, the influence of TMAO on cognition is unclear. In fact, there is much controversy as to whether TMAO promotes a positive or detrimental effect on the brain.

Both experimental [161–163] and clinical [164–166] studies suggest high levels of TMAO may be causally linked to cognitive decline. Vogt and colleagues discovered an increase in CSF TMAO in AD patients in comparison to controls, suggesting the metabolite may contribute to decreasing neurological function [147]. However, a recent Mendelian randomisation study disputes this relationship [167].

The mechanisms by which TMAO may contribute to cognitive decline remain broad and unclear. TMAO reportedly modulates lipid and hormonal homeostasis [147], encourages platelet hyperreactivity via the enhancement of stimulus-dependent release of calcium ions [168], modifies cholesterol and sterol breakdown, reduces reverse cholesterol transport [169], and increases endothelial dysfunction through the induction of the NLRP3 inflammasome [170]. Rodents fed supraphysiological doses of TMAO also suggest the metabolite promotes neuronal senescence, oxidative stress, mitochondrial dysfunction and prevents mTOR signalling [161]. Furthermore, TMAO is known to upregulate macrophage scavenger receptors and induce CD68 expression [169, 171], a marker known to correlate with cognitive impairment in rodents [172].

High circulating TMAO may also promote neuroinflammation, a recognised mediator of cognitive ageing and neurological function [173, 174], by increasing brain NF-κB and proinflammatory cytokines, thereby promoting proinflammatory signalling pathways [164]. Brunt and colleagues suggested that elevated TMAO in plasma and the brain can stimulate astrocytes, neuroinflammation and reduce cognitive function, especially in the subdomain of memory [164]. High circulating concentrations of TMAO also downregulated the antioxidant enzyme methionine sulfoxide reductase A in the hippocampus of aged rats with induced cognitive impairment by sevoflurane exposure [175]. This downregulation is suggested to sensitise the hippocampus to oxidative stress, promoting microglial mediated neuroinflammation and cognitive impairment. Collectively, studies indicate a detrimental effect of TMAO when modulated above physiologically relevant concentrations.

In line with this, reducing TMAO has been shown to alleviate cognitive impairment. 3,3-Dimethyl-1-butanol, an inhibitor of microbial TMA formation, reduced cognitive decline, long term potentiation and pathological deterioration in AD transgenic mice [162]. Similarly, the probiotic *L. Plantarum* decreased circulating TMAO levels, alleviating cognitive impairments and pathological deterioration, exhibiting the potential modulation of the gut microbiome for therapeutic benefit [176].

In contrast to the substantial evidence supporting a detrimental effect of TMAO upon the brain, several studies suggest TMAO may exert a neuroprotective effect when within normal physiological ranges (plasma levels ~ 0.5–5 µM). Hoyles and colleagues, using a mixed in vitro endothelial cell culture and in vivo rodent model approach, discovered that TMAO can enhance and protect BBB integrity through modulation of the actin cytoskeleton and tight junctions [177]. Here, administering TMAO reduced paracellular permeability, likely due to an increase in annexin A1 expression. TMAO, therefore, may promote BBB function and help protect the brain from an influx of cytotoxic molecules. Interestingly, TMA, the precursor to TMAO, was found to have a deleterious effect on endothelial barrier integrity in rodents, inducing actin stress fibre formation and leading to increased presence in the CNS [178].

TMAO is a naturally occurring osmolyte and as such has been found to stimulate TAU-induced tubulin assembly in vitro [179]. TMAO, therefore, can promote and enhance microtubule assembly in hyperphosphorylated and most mutant TAU proteins, decreasing microtubule disassembly and neuronal death; two hallmark features of AD [180]. TMAO overcomes functional deficits caused by phosphorylation by lowering the critical concentration of tubulin required for assembly [181], with assembly occurring at a faster rate than wild-type TAU [180]. Therefore, as an osmolyte, and with its ability to favourably hydrate partially denatured proteins, TMAO has been suggested as a potential therapeutic approach in AD and other protein misfolding conditions [182].

Collectively, it seems plausible that TMAO affects the brain in a dose-dependent manner, as within a physiologically relevant range, TMAO possess neuroprotective potential. However, interpreting the relationship between systemic TMAO and cognition is further complicated by studies indicating wide inter and intra-individual variations in circulating TMAO levels [183]. TMAO concentrations vary with age [184], diet [169] and cholic acid levels (a BA known to induce FMO3 expression via FXR activation [185]); factors often not accounted for in association studies. In fact, plasma TMAO concentrations have been found to mirror an individual’s intake of whole grain, fish and vegetables [186]. TMAO levels are also influenced by renal clearance, as glomerular filtration rate is inversely related to plasma TMAO concentrations [159]. As a result, changes in plasma TMAO may be a consequence of an accumulation of factors unrelated to cognitive decline [187].

### TMAO as a risk factor of cognitive decline

Due to TMAO’s high association with atherosclerosis and cardiovascular disease, TMAO has been considered a risk factor of vascular dementia [188]. However, a data-driven, hypothesis-free computational analysis into microbial metabolites and AD identified TMAO as one of the top potential biomarkers of neurodegeneration, successfully predicting changes in memory and fluid cognition in ageing individuals [189]. These results show promising potential for use of TMAO as a risk factor of cognitive decline. However, the current contrasting evidence surrounding the relationship necessitates further in vivo investigation.

## Amino acid-microbiota-derived metabolites

### Tryptophan

Tryptophan is an essential aromatic amino acid that cannot be synthesised by animal cells [190]. Humans, therefore, need to attain tryptophan through dietary sources such as fish, milk and chicken or, if vegetarian, seeds, soybeans and peas [191, 192]. Tryptophan is a biosynthetic precursor to numerous microbial and host metabolites, making it essential to human health [190]. Approximately 4–6% of tryptophan reaches the colon where gut microbiota metabolise it into a wide variety of molecules (Fig. 2), thereby limiting the availability of tryptophan for the host [193]. Evidence for the involvement of microbiota in tryptophan metabolism comes from GF mice, who display increased plasma tryptophan levels which are normalised after conventionalisation [62, 194].

Previous experimental reports implicate tryptophan and its derivatives in modulating human health and neurological function [195]. Gut microbiota can directly and indirectly modulate two major tryptophan metabolism pathways, the serotonin pathway and the kynurenine pathway (KP), affecting the concentration of various cognitively relevant metabolites and neurotransmitters [196–198]. Conversely, the two pathways can negatively influence microbial proliferation and diversity [199]. Gut microbiota can also directly metabolise tryptophan into indole and its derivatives [200], which has also been associated with cognitive function [191].

#### The kynurenine pathway and cognitive decline

Around 90–95% of dietary tryptophan is metabolised by the KP, mainly taking place in the liver, forming the intermediates kynurenic acid, quinolinic acid, picolinic acid, 3-hydroxykynurenine (3-HK) and nicotinamide adenine dinucleotide, known as kynurenines [201]. Only tryptophan, 3-HK and kynurenine are known to readily cross the BBB. However, fluctuations in the systemic concentrations of these metabolites directly impacts KP metabolism in the CNS, including the synthesis of kynurenic acid and quinolinic acid in the brain [202]. Quinolinic acid, an endogenous neurotoxin, is known to activate N-methyl-D-aspartate (NMDA) receptors, increase neuronal activity, elevate intracellular calcium concentrations and modulate BBB integrity [203]. Quinolinic acid can also increase neuronal glutamate release whilst inhibiting its reuptake by astrocytes and inhibiting glutamate synthetase synthase (an enzyme playing a crucial role in the glutamate metabolism in astrocytes) to produce a cytotoxic response [204, 205]. Kynurenic acid, on the other hand, plays a neuroprotective role against quinolinic acid’s toxicity, acting as an antagonist on both glycine and glutamate modulatory sites of NMDA receptors at high and low concentrations respectively [198]. However, the abnormal build-up of kynurenic acid can induce glutamatergic hypofunction, possibly disturbing cognitive functioning [205].

Accumulating evidence implicates the KP in AD progression and inflammatory responses [206]. Increased plasma concentrations of the cytotoxic quinolinic acid (from 192 to 334 nM) and reduced concentrations of tryptophan (from 29.83 mM to 22.09 mM) and neuroprotective kynurenic acid (from 30.94 nM to 20.85 nM) has been associated with AD patients in comparison to healthy controls [207]. Unbalanced upregulation of the KP may trigger a degree of injury to the surrounding tissues, playing a role in neurodegeneration [208]. Previous studies have found an inverse relationship between KP activation and cognitive performance [209].

In a cognitively healthy population, increased inflammatory markers are related to poor cognitive performance [210]. In AD, indoleamine 2, 3-dioxygenase (IDO), the enzyme responsible for catabolising tryptophan into products that enter the KP, is stimulated through proinflammatory cytokine activity, including interferon-gamma (IFN-γ) [211], interleukin-12 (IL-12), interleukin-18 (IL-18) [212], and the Aβ 1–42 fragment [213]. Complex neuroinflammation in the CNS is linked with AD development. Microglia and astrocytes, which contain all of the enzymes necessary for the KP, are the primary effectors of neuroinflammation in AD [214]. The edge of senile plaques in the hippocampus of *post-mortem* AD brain tissue has the greatest amounts of IDO and quinolinic acid expressed by microglia and astrocytes [215]. Activated microglia are the main source of quinolinic acid throughout neuroinflammation [216]. Quinolinic acid produces hyperphosphorylation of TAU in human cortical neurones, cytotoxicity in astrocytes and neurons, astrocytic activation and astrogliosis [208, 217]. Together, these studies strongly suggest the involvement of IDO and KP metabolism in neuroinflammation and cognitive impairment.

Accordingly, the KP is a well-rationalised therapeutic target for improving cognition. Several proof-of-concept studies using known KP pathway modulators, such as the kynurenine monooxygenase (KMO) inhibitor JM6, prevents spatial memory deficits, anxiety-related behaviours, and synaptic loss in APP Tg mice [218]. In addition, the IDO-1 inhibitor, coptisine, decreases the activation of microglia and astrocytes in APP/PS1 mice, preventing neuronal loss and improving cognitive function [208]. However, the specific relationship between tryptophan depletion or supplementation and the modulation of KP intermediates remains unclear [219–221].

#### Serotonin pathway and cognitive decline

Approximately 3% of dietary tryptophan is required to produce serotonin (5-hydroxytryptamine (5-HT)) and melatonin [193]. 5-HT is primarily found in the GI tract, blood platelets and the CNS and is synthesised via a two-stage enzymatic reaction involving tryptophan hydroxylase and aromatic amino acid decarboxylase. Serotonin synthesised in the GI tract cannot cross over the BBB under healthy conditions [222]. Tryptophan, on the other hand, can enter the CNS via carrier proteins [223]. Therefore, the gut microbiota importantly regulates tryptophan availability for serotonin synthesis in the CNS.

Enzymes such as tryptophan hydroxylase and IDO balance the ratio of tryptophan metabolism via the KP and serotonin pathways [224]. A shift in tryptophan metabolism to the KP decreases the availability of tryptophan in the serotonin pathway, consequently reducing serotonin availability for the host [225]. Serotonin plays a vital role in behaviours requiring high cognitive demand [196]. Reductions in serotonin, therefore, are frequently linked with declines in learning, memory consolidation [226] and long-term memory [227]. As such, serotonin is associated with neurological disorders such as depression [228] and AD [229], resulting in treatment options such as selective serotonin reuptake inhibitors (SSRI) to increase 5-HT neurotransmission and improve mood in the context of depression. In rodents, administering tryptophan orally, thereby increasing 5-HT neurotransmission, was found to improve memory acquisition, consolidation and storage [230], whilst daily tryptophan injections improved spatial memory [231]. Together, this evidence strongly suggests a link between cognitive decline and tryptophan through changes in tryptophan metabolism.

#### Other tryptophan metabolites

Numerous studies have identified abnormal tryptophan metabolism in patients with cognitive decline [71, 191, 232]. Although most studies link this association with the KP and its intermediates, other tryptophan metabolites, such as indole and its derivatives, may play a role. Bacterial tryptophan catabolites tryptamine, skatole, indole, indole-3- acetic acid (IAA), indole-3- acrylic acid (IA), indole-3-aldehyde (IAld), indole propionic acid (IPA), indoxyl-3-sulfate (I3S) and indole-3-lactic acid (ILA) are ligands of the aryl hydrocarbon receptor (AhR) [233–238]. AhR is a transcription factor widely expressed by cells in the immune system and known to play a role in inflammation, a factor highly associated with ageing and cognitive decline [239]. Antibiotic-treated mice administered with indole, I3S, IPA and IAld were found to have reduced CNS inflammation via AhR activation in astrocytes [240]. Wei and colleagues discovered activation of the AhR by indole could promote neurogenesis in the adult mouse hippocampus [241]. Interestingly, this result was found to be ligand specific as kynurenine, another known AhR ligand, failed to replicate these findings.

Both in vitro and in vivo studies have associated indoles with enhancing intestinal barrier function by increasing gene expression associated with the maintenance of epithelial cell structure and function [242, 243], thereby decreasing the concentration of neuroactive products in circulation [79]. The activation of AhR also helps preserve epithelial barrier function by maintaining tight junction integrity [244]. IA may also have anti-inflammatory and anti-oxidative effects in LPS-activated human peripheral blood mononuclear cells (PBMCs) by reducing IL-6 and IL-1β secretion and activation of the NRF2-ARE pathway [245], a pathway suggested to ameliorate cognitive deficits [246, 247].

#### Tryptophan & derivatives as risk factors of cognitive decline

Although no research studies to date have exclusively investigated the use of tryptophan and its derivatives as a risk factor of cognitive decline, many reports have highlighted the potential use of tryptophan pathway imbalances to reveal signs of early cognitive decline [248]. Kaddurah-Daouk and colleagues concluded from studying CSF of AD patients that changes in tryptophan, as well as methionine, tyrosine, and purine metabolism occurred in MCI and AD, suggesting its potential use as a risk factor of cognitive decline [232]. However, the authors concluded that these changes may not be detectable in plasma, as the amount to which metabolic changes in blood mirror fluctuations in CSF remains to be investigated. Nevertheless, plasma metabolic profiling revealed changes in tryptophan metabolism in early cognitive decline, along with alterations in progesterone, lysophosphatidylcholine, L-phenylalanine, dihydrosphingosine and phytosphingosine [248]. Despite a lack of studies into the use of tryptophan and its derivates as a risk factor of cognitive decline, these studies highlight the possible future use of metabolomic profiling to detect early changes.

### GABA

Through either direct access via the circulatory system, or via other communication routes, microbial metabolites may have the capacity to interfere and impact the function of the CNS [249, 250]. GABA is the main inhibitory neurotransmitter in the human brain and other parts of the body [251] and is reportedly unable to cross the BBB, although this statement is disputed [252]. This molecule was recently shown to be both a product of bacteria in the gut [250, 253, 254] and an important substrate for other gut community members [255]. It was also shown to have activity in rodent models of anxiety and visceral pain [256]. Bacterial strains, such as *L. rhamnosus,* can also modify GABA receptor expression and concentrations of glutamate (a precursor to GABA) and GABA in the brain [257].

### Other bacterial amino acid metabolites

Amino acids present in dietary protein serve (particularly if overconsumed) as a fermentation substrate for bacteria in the large intestine. *P*—Cresol is the product of the microbial conversion of tyrosine, notably by the bacteria from the *Coriobacteriaceae* or *Clostridium* genera [258]. *P*—Cresol is a known uremic toxin and therefore can be further conjugated with sulphate by host cells to form *p*—cresyl sulphate (PCS) as part of the detoxification mechanism, promoting the removal of the metabolite by the kidneys.

*P*—Cresol is known to increase endothelial permeability in vitro through modulation of the actin cytoskeleton and adherens junctions [259], decreasing the gut’s barrier function. In the brain, *p*-cresol has been found to modulate dopamine turnover in Autism Spectrum Disorder BTBR mice, significantly increasing anxiety-like and hyperactive behaviours [260]. *p*-Cresol’s derivative, PCS, has been detected in the CSF of PD patients, suggesting the metabolite may cross the BBB and have a pathogenic effect in the CNS [261]. Although, this relationship may in part be due to the increased permeability of the BBB seen in PD [262]. PCS has been linked with cell death and dysfunction through oxidative stress, inflammation, impairment of mitochondrial dynamics and vascular disruption [263–266]. Moreover, PCS administration in mice with nephrectomy contributed to neurological dysfunction through impairment of cell survival and neurogenesis, supporting its potential role in cognitive decline [267].

Imidazole propionate (ImP) has recently been uncovered as a microbially produced metabolite derived from the amino acid histidine [268]. Elevated serum concentrations of ImP are associated with low bacterial gene richness [269], a factor previously linked to low-grade inflammation, metabolic and inflammatory disorders [270]. ImP is also associated with a type 2 diabetes-related microbiome, stimulating impaired glucose metabolism through the initiation of the p38γ-mTOR1-S6K1 signalling pathway [268, 269, 271]. Type 2 diabetes is a well-characterised risk factor for dementia, with a 1.5–2.5-fold increase in dementia risk, suggesting an association between ImP, the gut microbiome and cognitive decline [272, 273].

## Other emerging microbial-derived metabolites

During digestion, nutrients and bioactives (proteins, amino acids, polysaccharides, fibres, fats, polyphenols, etc.) are catabolised into host-derived and bacterial metabolites that have the ability to interact with the host’s cells and the resident gut microbiome [256, 274]. This continuous process results in the production of a wide array of chemicals representing a wealth of chemical classes. Dietary proteins are broken down into potentially active peptides [256] that are further transformed into bacterial products such as neurotransmitter amino acids like glutamate, glycine, aspartate, serine and GABA or polyamines [250, 275, 276]. Aromatic amino acids (tryptophan, tyrosine, and phenylalanine) and polyphenols yield a myriad of compounds during catabolism leading to the formation of simpler structures containing at least one phenol ring (phenols) which can then be further transformed by the host (sulfation, glucuronidation) before re-entering circulation [256, 274]. Additionally, dietary choline and niacin are substrates for the synthesis of molecules essential for cellular function in the brain namely acetylcholine and nicotinamide adenine dinucleotide (NAD +) precursors [277–279], some of which have recently been shown to be synthesised by the gut microbiota [250, 254, 275, 278, 280]. These recent developments provide further evidence of the microbiota’s role in the production of beneficial signalling molecules that contribute to the maintenance of homeostasis during the ageing process.

As described earlier in the review, the BBB selectively allows circulating solutes to enter the CNS. Polyamines, polyphenols and some of their products (3-(3’-hydroxyphenyl)propionate and 3-hydroxybenzoate) [281] have been shown to cross the barrier even though the transfer seems somewhat limited [282–286]. Meanwhile, nicotinamide and niacin, both precursors for NAD + , a coenzyme essential for the maintenance of the CNS, have the capacity to freely cross the BBB [278, 287]. More research on the topic is needed as the knowledge regarding their transport across BBB is in its infancy and partly based on in vitro models [282, 286, 288]. The question of whether the potential activity of those molecules on brain functions is either direct or based on interactions with peripheral systems remains open [289].

### Short-chain fatty acids

Short-chain fatty acids (SCFAs) are small organic compounds primarily formed from microbial anaerobic fermentation of dietary fibres in the cecum and colon [290]. Accumulating evidence suggests SCFAs can attenuate cognitive decline, however, the underlying mechanisms remain unclear [291]. Recent studies suggest SCFAs can cross the BBB via monocarboxylate transporters present in endothelial cells within the brain tissue [292]. In fact, the uptake of SCFAs into the brain has formerly been exhibited in rodents following the injection of ^14^C-SCFAs into the carotid artery [293]. However, as well as crossing the BBB, SCFAs may also help preserve its integrity. GF mice with reduced SCFA levels were found to exhibit increased BBB permeability due to a reduction in the expression of tight junction proteins [294]. This BBB dysfunction was later reversed following conventionalisation with pathogen-free microbiota and monoculture strains producing SCFAs. Furthermore, in a rodent model of traumatic brain injury, the administration of sodium butyrate prevented BBB breakdown and promoted neurogenesis, highlighting a key role for SCFAs in not only maintaining CNS homeostasis but also possibly in preventing or reducing neural decline [295].

Select SCFAs can also manipulate epigenetic mechanisms, including DNA methylation, histone modification and their interactions, which may influence age-related cognitive changes [296]. Butyrate has been widely investigated due to its roles in receptor signalling and metabolic regulation. However, pharmacological studies also highlight butyrate as a histone deacetylase inhibitor, capable of increasing histone acetylation and inducing the expression of neurotrophic and anti-inflammatory genes [297, 298]. Accumulating evidence also suggests a role for butyrate in modifying DNA methylation [299–301].

In the CNS, SCFAs have also been linked to reducing neuroinflammatory processes important for shaping brain function. Sodium butyrate has been linked to a decrease in microglial activation and pro-inflammatory cytokine secretion [297, 302]. Rodents supplemented with dietary acetate had a decrease in neuroglial activation by reducing the expression of pro-inflammatory cytokines and modulating brain histone acetylation [303]. Likewise, acetate also modulated inflammatory cytokines and signalling pathways in astrocyte primary culture [304]. In vivo and in vitro sodium butyrate administration was observed to have an anti-inflammatory role via protein kinase B (Akt)-RhoGTPase signalling and histone deacetylase inhibition, stimulating structural and functional changes in microglial towards a homeostatic profile [305]. SCFAs may also improve brain hypometabolism, a known contributor to neuronal dysfunction and AD, by providing an alternate substrate for energy metabolism [306, 307] highlighting a further potential method to mitigate and protect against neuroinflammatory processes. Nevertheless, the precise signalling underlying SCFA's influence within the CNS remains unclear, however, the inhibition of histone deacetylase has been put forward as the primary mechanism [308].

Select SCFAs may also moderate AD progression [309]. For example, valeric acid, butyric acid and propionic acid have been found in vitro to interfere with protein–protein interactions necessary for Aβ assemblies, potentially reducing the formation of toxic aggregates [290]. Yet, it remains unclear if SCFAs produced in the GI tract can play a role in protein misfolding in vivo [290]. However, Colombo and colleagues found GF AD mice display reduced circulatory SCFA concentrations and Aβ deposition, yet when supplemented with SCFAs, show an increase in Aβ plaque deposition, suggesting SCFA mediation [309]. In line with this, a clinical study into elderly individuals with ranging cognitive performance found an association between SCFA levels in the blood and brain amyloid deposition [310].

*APOE* genotype, the largest genetic risk factor of AD, has been associated with the composition of butyrate-producing microbiota in the gut [311]. Faecal samples from AD patients typically consist of an abundance of SCFAs, particularly butyrate-producing bacteria [312]. However, currently, there is no comparison of SCFA concentrations in age-matched healthy controls and therefore its use as a risk factor of cognitive decline is limited. This may in part be due to SCFA's volatile nature, making the compound difficult to detect in human samples, and current research also demonstrating low reproducibility. Notably, participant diet is rarely incorporated when quantifying SCFAs in research studies. Yet, the quantity and type of ingested fibre are known to have a large influence on microbial composition and, therefore, the concentration and type of SCFAs produced [306, 313]. Faecal SCFA concentrations also cannot fully signify production rates or accurate concentrations of SCFAs present in the colon, as significant percentages of SCFAs are immediately consumed locally in the gut [314, 315]. Changes in SCFA faecal concentrations, therefore, may be the result of either a fluctuation in its production or colonic absorption. Consequently, as of present, the knowledge on using SCFAs as a risk factor of cognitive decline is extremely limited.

### Acetylcholine

Acetylcholine (ACh) is a common cholinergic neurotransmitter in the central and peripheral nervous systems. In the periphery, ACh can be produced from choline by numerous bacteria, including *Lactobacillus plantarum, Bacillus subtilis, Escherichia coli,* and *Staphylococcus aureus* [316, 317]. Within the microbiota-gut-brain axis, ACh can modulate intestinal motility, secretion and enteric neurotransmission. essential for the transmission of excitatory signals between neurons. Its dysregulation is closely linked with AD [318]. ACh cannot cross the BBB. Therefore, choline availability in the periphery importantly modulates the concentration of ACh in the CNS [319].

### Dopamine

Dopamine is the leading catecholamine neurotransmitter in the mammalian CNS, playing a key role in a broad spectrum of cognitive abilities, including working memory, planning, selective attention abilities, motivation and reward processing [320, 321]. Dopaminergic transmission abnormalities have been linked to cognitive decline and numerous CNS disorders [reviewed by [322]]. Dopamine itself cannot cross the BBB. However, its precursor molecule L-3,4-dihydroxyphenylalanine (L-DOPA) can be transported across the BBB by large neutral amino acid transporters (LAT1) expressed on endothelial cells [323].

One approach to investigating the involvement of gut microbiota and their metabolites on cognitive decline is through the use of broad-spectrum antibiotics to induce gut dysbiosis by preventing the growth of select microorganisms. Administering an antibiotic cocktail of ampicillin, vancomycin, neomycin, metronidazole, and amphotericin B to the drinking water of male Swiss mice increased concentrations of L-DOPA and homovanillic acid (HVA), a dopamine-derived metabolite, in the amygdala in comparison to control mice [66]. However, no significant changes in dopamine levels were detected. Similarly, Hoban and colleagues observed increased concentrations of L-DOPA in the prefrontal cortex and hippocampus of adult male Sprague–Dawley rats after supplying an antibiotic cocktail of ampicillin, vancomycin, ciprofloxacin, imipenem, and metronidazole for 42 days. Together, these studies suggest antibiotic-induced dysbiosis can impact dopamine neurochemistry in the rodent brain.

As discussed earlier in this review, one mechanism in which intestinal bacteria can communicate with the brain is via stimulation of the vagus nerve. Interestingly, Han and colleagues found stimulation of vagal afferent fibres from the upper intestinal tract can promote dopamine release in the brain of mice [324]. Dopamine can also be synthesised in the intestinal lumen by gut microbes [325]. Indeed, gut microbes belonging to the genus *Prevotella, Bacteroides, Lactobacillus, Bifidobacterium, Clostridium, Enterococcus*, and *Ruminococcus* have been suggested to modulate dopaminergic activity and influence Parkinson’s disease (PD) pathophysiology (reviewed by [326]). Gut microbiota can also increase luminal dopamine bioavailability through enzymes such as β- glucuronidase [325] and tyrosine decarboxylase [327], demonstrating a key role of the gut microbiota in modulating peripheral dopamine levels. Interestingly, plasma L-DOPA levels were found to be significantly increased in probable AD patients in comparison to controls, whereas dopamine concentrations were decreased [328].

### Polyphenols

Both plant-based foods, rich in polyphenols, and dietary proteins are substrates for colonic bacteria which produce phenolic compounds that potentially benefit human health [256, 274, 329–331]. The current research on the impact of beneficial and harmful microbial phenolic compounds 3-(3’-hydroxyphenyl) propionate, 3-hydroxybenzoate, indoxyl sulfate, p-cresol sulfate on the brain is at an early stage but determining the role of those products on the gut-brain axis is a promising field of research [256, 274, 281, 330]. Frolinger and colleagues have recently demonstrated a link between polyphenolic products produced by gut microbiota and cognitive resilience in rats [332] and Esteban-Fernández et al. showed that 3-hydroxyphenylacetic acid and other microbial-derived phenolic compounds have a neuroprotective effect on a human neuroblastoma cell line [333]. Metabolomics on circulating metabolites also correlated levels of catabolites of the phenylalanine and tyrosine pathways to poorer mini-mental state examination (MMSE) scores in a cohort of hypertensive patients [334]. Mostly driven by in vitro and animal-based studies, research on the effect of phenolic compounds are nonetheless accruing evidence that microbial phenolic compounds could play a role in brain metabolism [286].

### Polyamines

Polyamines were first described in 1677 by Antonie van Leewenhoek who reported the presence of crystals in human semen [284]. It was much later in 1924 that Dudley and colleagues characterised one of their components, isolating spermine from bovine brain [284]. Polyamines are small molecules essential to cell growth and ubiquitous to all life forms. Most of the polyamine pool is bound to RNA conferring an important role to polyamines in stabilising this molecule and contributing to the process of its translation [284]. Putrescine, spermidine and spermine are synthesized by plants, mammals and bacteria and represent the most abundant polyamines found in tissues [335–337].

Levels of polyamines found in mice decline with brain ageing [65, 338] but in humans, only spermidine levels seem to change over time reaching their highest level at 40 years of age and remaining at similar levels thereafter [336]. Elevated levels have been reported in the brain from AD patients, where increased ornithine decarboxylase activity was found to be associated with AD processes [339].

They are abundant in food, quickly absorbed and distributed to all body tissues [340]. The polyamine content in the lower part of the intestine however is considered to be mostly of microbial origin [337]. Sustained circulating levels at an older age have been associated with enhanced longevity and the prevention of age-associated disease [340, 341]. Conversely, lower spermidine levels were found in blood from AD patients when compared to healthy individuals [342], a characteristic that was associated with lower MMSE scoring in another study conducted on older subjects in nursing homes [343].

Preserving adequate levels of polyamines could represent a valuable approach to maintaining the optimal functioning of cell metabolism and the prevention of chronic illnesses. Supplementation could be achieved by a regular intake of a polyamine-rich food diet or synthetic polyamines, or by the provision of microbial polyamine synthesis with probiotic supplements [344]. A recent study highlighted olive oil, fruits, cheese, and seafood as good sources of polyamines and that a steady intake may have a role in prolonging human life. The authors speculated that the mechanism involved could be a capacity for polyamines to counteract mild chronic inflammation and confer beneficial effects on vascular function [340]. Another study reported an association between spermidine intake estimated with a self-reported food frequency questionnaire and cortical thickness and hippocampal volume in older adults [345].

A study on mice fed arginine, a precursor for the synthesis of polyamines [276, 337] and probiotics LKM512 showed that long term administration offered protection against age-induced memory impairment via a mechanism involving the production of polyamines by microbiota [341]. The putative protective properties of polyamines are inhibition of cytokines release, inhibition of reactive oxygen species (ROS) production [346, 347], an impact on T-cell function and the maintenance of synaptic plasticity through the prevention of demyelination [347], thus presenting a defence against events that embody hallmarks of neurodegeneration [348, 349]. Polyamines also have the capacity to induce cytoprotective autophagy, a process involving the degradation of damaged organelles and biological debris [344, 350, 351]. They have a significant role in the maintenance of mitochondrial metabolic function. Indeed, spermidine is needed to chemically modify eukaryotic initiation factor 5A (eIF5A), an important enzyme involved in TCA cycle maintenance and electron transport chain in macrophages [352], highlighting an important role for this polyamine in the regulation of mitochondrial metabolism as any reduced activity can lead to neuroinflammation and neurodegeneration [348, 349].

As mentioned earlier, host bacterial production of polyamines was recently shown to delay senescence in mice [341]. Although the exact mechanisms were not elucidated, the authors speculated that autophagy [350, 351] may play a role in the preservation of memory capacity in ageing. This is further reinforced by recent studies on mice which showed that supplementation by spermidine and spermine may delay brain ageing and alleviate AD pathology via mechanisms involving autophagy, promotion of ATP, reduction of ROS [353, 354] and inflammation [355]. Maglione and colleagues showed that spermidine offered protection from synaptic alterations in the hippocampus of ageing mice extending their lifespan with a late treatment (starting at 18 months) [356].

The research on both autophagy and polyamines and cognitive health, which is getting traction, has recently translated into human trials. A long-term spermidine-rich treatment (dosage: 1.2 mg/day) was given to participants at risk of developing AD and found to be safe and well-tolerated [357]. This 3‐month randomized, placebo-controlled, double‐blind Phase II trial was shown to moderately improve the memory performance and to enhance the mnemonic discrimination ability of the treated individuals compared to the placebo-treated group [358]. The authors have designed a new trial using the same treatment that will expand the intervention period to 12 months and will include a larger cohort (*n* = 100 as opposed to *n* = 30) and a follow-up assessment 18 months after the start of the study [359]. In parallel, another group supplied older adults in nursing homes with spermidine added to bread for 3 months and evaluated the cognitive performance of the subjects with the CERAD-Plus test which consists of seven tests including an MMSE, a learn, recall and recognize a word list and phonemic fluid [360]. They reported a significant correlation between an intake of spermidine and improvement in cognitive performance, particularly in subjects with mild and moderate dementia. Their preliminary results offer hope for the possible mitigation of cognitive decline by enabling sustainable levels of polyamines in the body.

### Nicotinamide

Energy and niacin and nicotinamide pathways are under tight homeostasis as shown by a lack of change in ATP and nicotinamides levels in the brain of colonised ex germ-free mice [65]. There is evidence that the levels of these molecules which are essential for the development and maintenance of CNS neurons decline with age and in neurogenerative states [277]. Promising results from an AD animal model led to a 24-week double-blind, placebo-controlled randomized clinical trial of nicotinamide in subjects with mild to moderate AD [279]. Unfortunately, this study failed to show an improvement in cognitive function in those volunteers. A similar study provided a 10-week supplementation with nicotinamide riboside (NR) to older individuals with MCI [361]. This trial resulted in demonstrating a positive effect on certain functions in the brain and frailty measures but like in the previous study, ultimately ended in a lack of change in cognitive measures [361]. This illustrates the complexity of translating results from animal studies to human trials with the dose, duration of the supplementation and environmental factors affecting the likelihood of a successful outcome. Nonetheless, recent studies showed that the gut microbiota can assist in the production of nicotinamide and other NAD + precursors [254] as demonstrated by Kim and colleagues who showed that treatment with nicotinamide mononucleotide in mice not only led to the microbial production of the deamidated product nicotinic acid mononucleotide, but also tripled the endogenous levels of NR, showing an important connection between the gut microbiome and the niacin and nicotinamide pathway [280].

### Vitamin K

Vitamin K is a vital micronutrient that can be derived directly from our diet (phylloquinone) or intestinal microbiota (menaquinone) [362]. Vitamin K’s role is well-defined in blood coagulation and its beneficial effects on myelin integrity in the brain [363, 364]. Recent studies outline a positive relationship between vitamin K levels and cognitive performance [365, 366], and the administration of vitamin K antagonists to rats alters cognitive performance [367]. Increased dietary vitamin K intake is linked to a decrease in subjective memory complaints in an elderly cohort [368], whilst low concentrations of vitamin K in the blood have been correlated with the *APOE-ε4* allele; the largest genetic risk factor of AD [363]. However, the direct relationship between microbial-derived vitamin K and cognition, and hence its use as a risk factor of cognitive decline, is yet to be uncovered.

## Conclusions and future directions

**Fig. 3:**
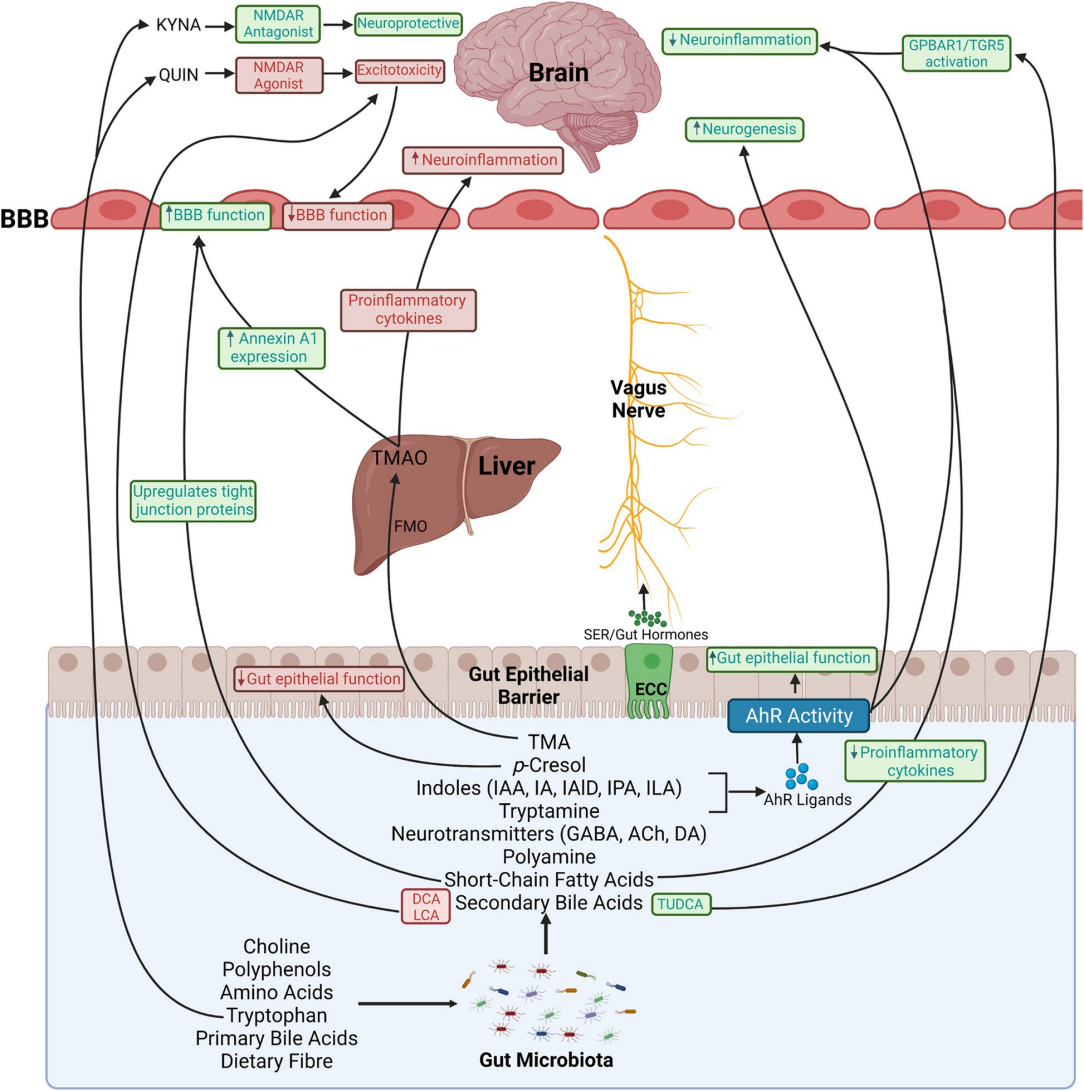
Key potential pathways through which microbial-derived metabolites influence cognitive function. An illustration of the main underlying mechanisms linking microbial metabolites and the brain. Dietary-derived precursor molecules can be metabolised by gut microbiota to form bioactive metabolites. These microbial-derived metabolites can influence gut permeability, blood–brain barrier function, neuroinflammation, vagus nerve activation, neurogenesis and excitotoxicity affecting the regulation of the microbiota-gut-brain axis and cognitive function. The green colour highlights a protective and beneficial effect, whereas red indicates a detrimental effect. Acronyms: BBB: blood–brain barrier; DCA: deoxycholic acid; ECC: enterochromaffin cells; FMO: flavin-containing monooxygenase; GABA: γ-aminobutyric acid; IA: indole-3- acrylic acid; IAA: indole-3- acetic acid; IAld: indole-3-aldehyde; ILA: indole-3-lactic acid; I3S: indoxyl-3-sulfate; KYNA; kynurenic acid; LCA: lithocholic acid; NMDAR: N-methyl-D-aspartate receptor; QUIN; quinolinic acid; TMA: trimethylamine; TMAO: trimethylamine N-oxide; TUDCA: tauroursodeoxycholic acid

The concept of microbial-derived metabolites influencing cognitive decline is gaining traction, with implications in the field of neuroscience, metabolomics and hepatology. However, due to the complexity of this relationship, the specific myriad of mechanisms responsible remain largely unknown, whilst defined roles of individual metabolites are only characterised for a select few (for a summary see Fig. 3). Therefore, amid this ambiguity, there remains a real need for additional research to highlight and validate key pathways, metabolites and mechanisms to further elucidate the influence of the microbiota-gut-brain axis on cognition [189].

There remain many challenges facing this growing field. Firstly, a lack of specificity limits our ability to distinguish between host vs microbiota-derived metabolite contribution as particularly if there is known co-metabolism, true microbial involvement may be masked or exaggerated. Secondly, as demonstrated by TMAO [362], some inconsistencies still exist among certain metabolites under context-specific vs dose-specific conditions. This may in part be due to heterogeneity between studies, with variations in study designs, methods of assessing cognitive performance and/or quantifying metabolites. As a result, further research ought to be collated via a more standardised methodology to increase comparability. Thirdly, the influence of the microbiome on cognition is not the totality of microbial metabolites produced in the gut as the varying capabilities of these metabolites to penetrate the BBB play a key role [79]. Consequently, the mechanisms used by many metabolites to cross the BBB are still unknown and some may even be synthesised de novo. Fourthly, from a translational perspective, the described research has largely been conducted in animals. Establishing whether these findings translate to humans will be crucial yet challenging due to the greater complexity and environmental exposure humans encounter, in turn shaping each individual’s microbiome [369]. Finally, understanding these highly complex systems, particularly as we move more towards human studies, requires the continued advancement of computational and statistical methods to obtain and implement multi-omics and longitudinal data necessary for a comprehensive approach [330]. Together, these challenges render it difficult to outline specific host-microbiota interactions in a mechanistic manner, which is needed to advance the field past associations towards implementable microbiota-driven targets.

Nevertheless, the wealth of association studies highlight a positive future for the use of microbiota-derived metabolites as risk factors of cognitive decline [370]. Future studies should progress using robust and replicable metabolic phenotyping across various stages of cognitive decline in humans. Recent advancements using this approach are underway, utilising metabolic phenotyping of urine [371] and blood [153, 372] to predict incipient AD with high degrees of accuracy. However, several studies using comparable approaches have not been able to replicate these findings [373, 374]. This may be due to intrinsic difficulties surrounding the heterogeneity of cognitive decline seen in neurodegenerative diseases and the variety of analytical methods used in metabolic profiling, ranging from ^1^H-NMR, LC–MS/MS, GC–MS, UHPLC-MS and CE-MS [371]. Hence, currently, the literature is too scarce to support the implementation of metabolite-derived risk factors in clinical practice.

In conclusion, although significant work remains to fully understand the role of microbial-derived metabolites as key mediators of cognitive decline, identifying modifiable factors that promote healthy ageing and cognition will have vital clinical implications in today’s growing elderly population, whilst also helping to identify novel underlying mechanism.

## Data Availability

Not applicable.

## Abbreviations

5-HT: Serotonin (5-hydroxytryptamine)
ACh: Acetylcholine
AD: Alzheimer’s disease
AhR: Aryl hydrocarbon receptor
Aβ: Amyloid-β
BA: Bile acids
BBB: Blood–brain barrier
BDNF: Brain-derived neurotrophic factor
CA: Cholic acid
CDCA: Chenodeoxycholic acid
CNS: Central nervous system
CSF: Cerebrospinal fluid
CYP27A1: Sterol 27-hydroxylase
CYP46A1: Cholesterol 24-hydroxylase
CYP7A1: Cholesterol 7α-hydroxylase
DCA: Deoxycholic acid
DMN: Default mode network
ECC: Enterochromaffin cells
FMO1/3: Flavin-containing monooxygenase 1/ 3
GABA: γ-Aminobutyric acid
GF: Germ-free
GI: Gastrointestinal
GUDCA: Glycoursodeoxycholic acid
HVA: Homovanillic acid
IA: Indole-3- acrylic acid
IAA: Indole-3- acetic acid
IAld: Indole-3-aldehyde
IDO: Indoleamine 2, 3-dioxygenase
ILA: Indole-3-lactic acid
ImP: Imidazole propionate
IPA: Indole propionic acid
I3S: Indoxyl-3-sulfate
KP: Kynurenine pathway
LAT1: Large neutral amino acid transporters
LCA: Lithocholic acid
L-DOPA: L-3,4-Dihydroxyphenylalanine
LPS: Lipopolysaccharide
MCI: Mild cognitive impairment
MMSE: Mini-mental state examination
MRI: Magnetic resonance imaging
NMDA: N-methyl-D-aspartate
NR: Nicotinamide riboside
PBMCs: Peripheral blood mononuclear cells
PCS: *p*-Cresyl sulphate
PD: Parkinson’s disease
PET: Positron emission tomography
ROS: Reactive oxygen species
SCFAs: Short-chain fatty acids
TLR: Toll-like receptor
TMA: Trimethylamine
TMAO: Trimethylamine N-oxide
TUDCA: Tauroursodeoxycholic acid
